# Negative response to immunotherapy in dMMR or MSI-H gastric cancer with APC and PTEN mutations: a case report

**DOI:** 10.3389/fonc.2024.1484802

**Published:** 2024-11-28

**Authors:** Jiang Liu, Xiumei Zhang, Qun Ren, Chuanjun Song, Jianhe Yu, Yin Cai, Dadong Chen

**Affiliations:** Department of Oncology, The Affiliated Xinghua People’s Hospital, Medical School of Yangzhou University, Xinghua, Jiangsu, China

**Keywords:** microsatellite instability high, mismatch repair-deficient, gastric cancer, immunotherapy, case report

## Abstract

**Background:**

Microsatellite instability-high (MSI-H) or deficient mismatch repair (dMMR) represents a distinct molecular phenotype observed in malignant tumors. These tumors typically exhibit high levels of programmed cell death 1 ligand 1 (PD-L1) expression and high tumor mutational burden (TMB), resulting in an enhanced response to immune checkpoint inhibitors (ICI) therapy. The emergence of ICI has transformed the therapeutic strategy of gastric cancer (GC). Immune checkpoint blockade significantly improves the survival of gastric cancer patients, especially those with MSI-H or dMMR. However, it’s worth noting that not all patients with MSI-H respond favorably to this treatment. It has been reported that factors such as tumor heterogeneity, alterations in the tumor microenvironment, and aberrant activation of tumor-related signaling pathways have been linked with resistance to ICI therapy.

**Case presentation:**

Here, we describe a case of dMMR and MSI-H GC with adenomatous polyposis coli (APC) and phosphatase and tensin homolog deleted on chromosome ten (PTEN) mutations that failed to respond to anti-PD-1 combined with anti-HER2 (human epidermal growth factor receptor-2) therapy and chemotherapy. We attempted to elucidate the underlying causes and mechanisms behind this lack of response, and to provide new insights into treatment options for these patients.

**Conclusions:**

Mutations of key genes within tumor-related signaling pathways and the infiltration of CD8^+^T cells in the tumor microenvironment may influence the efficacy of immunotherapy for MSI-H solid tumors.

## Introduction

Gastric cancer (GC) remains one of the leading and fatal malignant tumors with high heterogeneity and aggressiveness, ranking fourth for mortality and fifth for incidence globally (1). Unfortunately, more than 50% of patients are diagnosed with locally advanced and metastatic stages, eliminating the option for surgical resection and leading to a poor prognosis. Recently, ICI therapy has emerged as a new standard of treatment for advanced and metastatic GC, and has shown favorable clinical benefits in some populations (2–4). In addition, the combination of pembrolizumab, trastuzumab and chemotherapy revealed favorable clinical benefits as first-line therapy for HER2-positive advanced GC patients (5). However, the GC populations who benefit from immunotherapy are very limited in the real world.

GC with dMMR accounts for approximately 5% to 20% (6). Tumors with dMMR are particularly subject to mutations in repetitive DNA sequences, leading to high levels of microsatellite instability (MSI-H) (7, 8). KEYNOTE-177 phase III clinical study demonstrated that pembrolizumab significantly extended progression-free survival(PFS)compared to chemotherapy for MSI-H or dMMR metastatic colorectal cancer patients in the first-line setting (9). In addition to colorectal cancer, ICI therapy has also demonstrated significant efficacy in GC with MSI-H. Regardless of the line of therapy, MSI-H status predicts preferable survival outcomes in advanced gastric or gastroesophageal junction cancer patients treated with pembrolizumab (10). Nevertheless, there are a subset of MSI-H/dMMR GC patients who do not respond to it. Findings from a small sample demonstrated that low TMB and PTEN mutations, particularly mutations in the domain of phosphatases may be negatively associated with the response to anti-PD-1 therapy in patients with MSI-H/dMMR gastrointestinal tumors (11). Alterations in tumor cell intrinsic and extrinsic factors, as well as aberrant activation of tumor-related signaling pathways contribute to the underlying resistance mechanisms of ICI therapy (12). With the development of next-generation sequencing (NGS), genetic analysis of the MSI-H/dMMR phenotype of GC has hinted that certain gene mutations may lead to primary or acquired resistance to ICI therapy. In this case study, we present a GC case with dMMR/MSI-H status, HER-2-positive, as well as APC and PTEN mutations who suffered a negative response to anti-PD-1 therapy. Additionally, the potential causes and mechanisms of resistance were also tentatively explored.

## Case presentation

**Figure 1:**
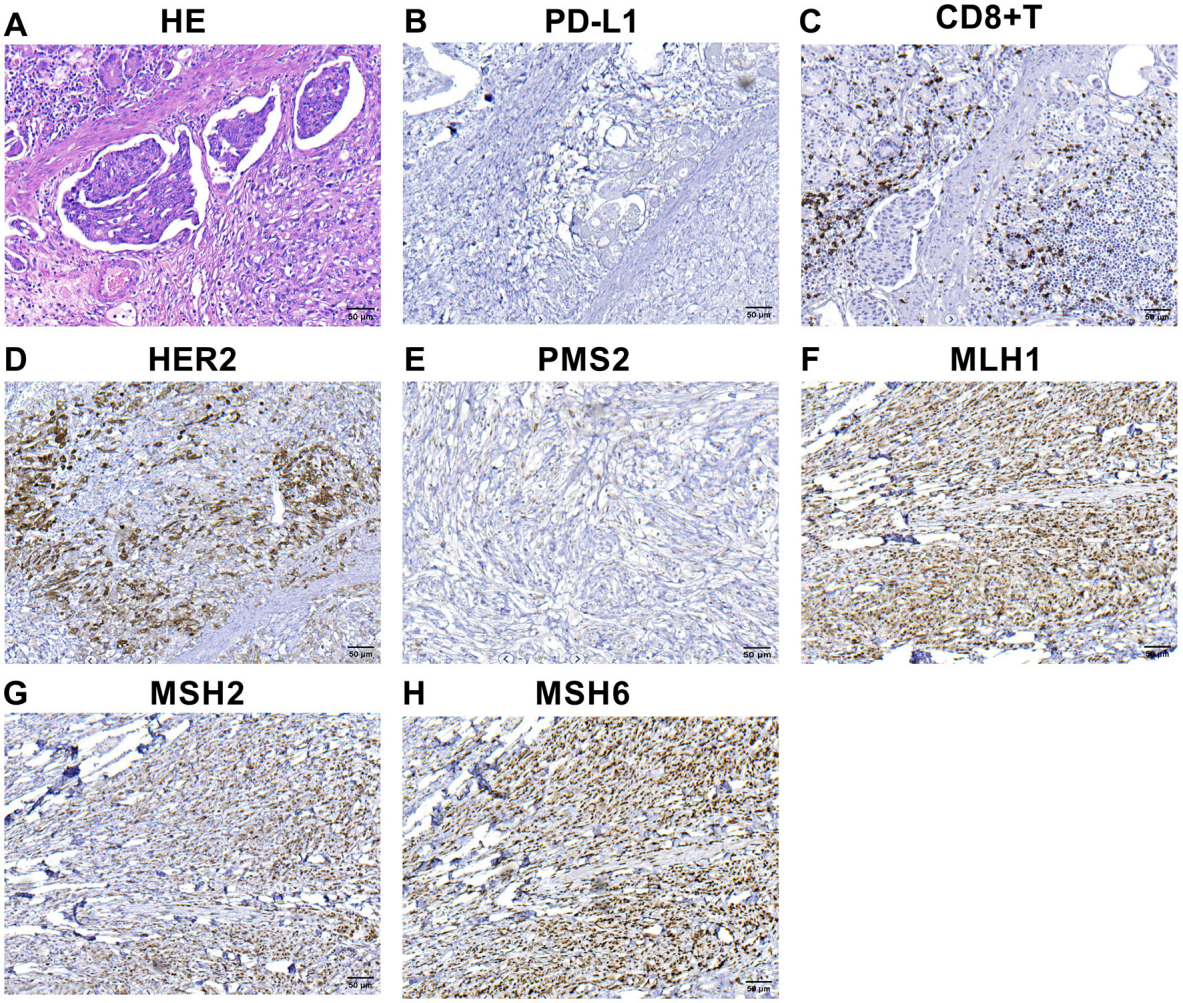
IHC and hematoxylin-eosin (HE) staining findings of primary tumor **(A–H)** HE staining of primary tumor **(A)**.The combined positive score of PD-L1 expression in primary tumor before treatment **(B)**. Infiltration of CD8^+^ T cells in the microenvironment of primary tumor before treatment **(C)**. HER2 expression in the primary tumor **(D)**. The expression of the protein PMS2 **(E)**, MLH1 **(F)**, MSH2 **(G)** and MSH6 **(H)**. MLH1, MSH2 and MSH6 were positively expressed, whereas PMS2 was lacking. The microscopic magnification of all images is 200.

A 65-year-old female patient was admitted to the hospital with upper abdominal pain and tarry stool for one week in October 2021. A gastroscopy examination revealed an ulcerative lesion in the gastric body. The gastroscopic biopsy confirmed adenocarcinoma of the gastric body, and computed tomography (CT) examination indicated no distant metastasis. This patient then underwent a radical total gastrectomy in our hospital. Hematoxylin-eosin (HE) staining of postoperative pathological tissue was shown in **Figure 1A**. Pathological examination of the surgical specimen revealed ulcerated poorly differentiated adenocarcinoma, classified as pT3N0M0, HER2-positive [immunohistochemistry (IHC) 3+, fluorescence *in-situ* hybridization (FISH) positive] and PD-L1-negative (IHC -), with a deficiency of PMS2 protein indicating dMMR (**Figures 1E-H**). Helicobacter pylori (H. pylori) was negative. There was no recurrence or metastasis in this patient’s postoperative baseline assessment. Postoperatively, the patient received six cycles of XELOX chemotherapy.

**Figure 2:**
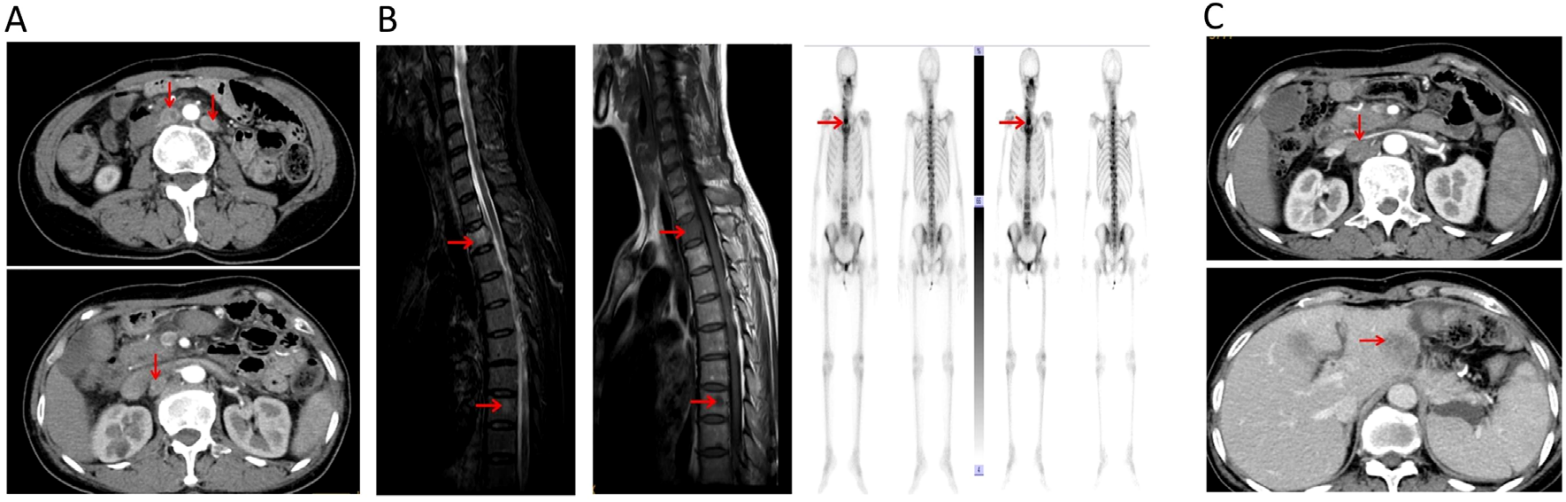
**(A)** Radiologic images of retroperitoneal lymph node metastases 17 months after operation. **(B)** Radiologic images of bone metastases after the first-line treatment. **(C)** Radiologic images of another retroperitoneal lymph node and liver metastases after the second-line treatment.

However, the patient experienced a recurrence in retroperitoneal lymph nodes 17 months after surgery (**Figure 2A**). The physical examination did not reveal any palpable superficial enlarged lymph nodes throughout the body. We reviewed the results of tumor tissue IHC, and found a deficiency of PMS2 protein (**Figure 1E**) and HER2-positive (**Figure 1D**). MSI-H status was confirmed further by PCR and NGS in this patient. The PCR test indicated that three single nucleotide sites (BAT25, BAT26, and D2S123) were changed. NGS also revealed a high TMB in both peripheral blood and tumor tissue, suggesting that this patient may benefit from ICI therapy. Based on the findings of KEYNOTE-811 clinical trial, the patient was subsequently treated with anti-PD-1(sintilimab, 200mg) in combination with anti-HER2 (trastuzumab, the first loading dose was 8 mg/kg, and the maintenance dose was 6 mg/kg) therapy and chemotherapy (S-1, 50mg, twice a day).

Unfortunately, after 3 months of the first-line treatment, this patient presented with back pain and weakness in both upper limbs. During the physical examination, no tenderness was elicited upon palpation of the back region. Emission Computed Tomography(ECT)and magnetic resonance imaging (MRI) confirmed metastatic bone destruction in the second and eighth thoracic vertebrae (**Figure 2B**). The patient’s second thoracic vertebra metastatic lesion was performed with local radiotherapy, synchronized with apatinib treatment. The patient’s back pain was significantly relieved at the end of radiation therapy. Subsequently, the patient initiated a second-line treatment regimen comprising anti-PD-1(sintilimab, 200mg) in combination with chemotherapy (albumin-bound paclitaxel, 200mg) and apatinib every three weeks.

**Figure 3:**
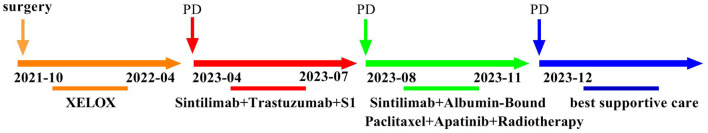
The entire treatment process of this patient.

However, after two months on the second-line treatment, this patient developed another retroperitoneal lymph node and liver metastases (**Figure 2C**). The physical examination did not reveal any palpable superficial enlarged lymph nodes throughout the body. Owing to the patient’s compromised physical condition, the decision was made to discontinue anti-tumor treatment in December 2023, and the patient transitioned to best supportive care. The entire treatment process of this case was shown in **Figure 3**.

## IHC test and NGS analysis

IHC assay was used to detect the expression level of PMS2, MLH1, MSH6, MSH2, as well as PD-L1-positive and CD8^+^T cells in the formalin-fixed, paraffin-embedded (FFPE) tumor tissues. For quantitative analysis, image Pro-Plus software was used to analyze the density of CD8^+^T cells and PD-L1-positive tumor cells. The number of CD8 ^+^ T cells within tumor tissues and stroma was 44 cells/mm^2^ and 268 cells/mm^2^, respectively. Notably, no PD-L1 positive tumor cells were detected. These data revealed that very few CD8^+^T cells were infiltrated in the tumor, and large numbers of CD8^+^T cells were infiltrated in the stroma (**Figure 1C**). Furthermore, negative expression of PD-L1 protein and a deficiency of PMS2 protein were observed in this case (**Figures 1B, E**).

The tissue DNAs and circulating tumor DNAs (ctDNA) were extracted using the GeneRead DNA FFPE Kit (Qiagen) and Qiagen DNA blood mini kit (Qiagen), respectively. The extracted DNAs were amplified, purified and analyzed using a panel (YuceOneTM Plus X, Yucebio, China). TMB was calculated based on non-silencing somatic mutations, including coding base substitutions and insertions or deletions. TMB-High (TMB-H) was defined as TMB > 20 muts/Mb. The FFPE –derived results disclosed three gene mutations with notable frequencies, including APC R1114*, APC R1450* and PTEN R233Q *. Conversely, the peripheral blood test results showed five gene mutations with high frequency, including PTEN K260T, EGFR R677C, APC R1114*, APC R1450*and KIT R135C. Besides, the results of TMB from tumor tissue and peripheral blood were 66.5 muts/Mb and 45 muts/Mb, respectively.

## Discussion

This case pertains to a patient with MSI-H GC presenting APC and PTEN mutations and exhibiting unresponsiveness to ICI therapy. This case presents notable characteristics encompassing PD-L1 expression, TMB, HER2 status, and specific genetic alterations in a MSI-H GC patient. We attempted to expound the causes for resistance to ICI therapy in this case. Two methods are usually used to screen MSI and MMR status. In this instance, IHC showed a deficiency of PMS2 protein, and the PCR test also revealed alterations at three single nucleotide sites (BAT25, BAT26 and D2S123), thereby affirming the presence of dMMR) or MSI-H traits in this patient.

The PTEN gene mutations observed in the tumor tissues differ from those in peripheral blood in our case. The potential reasons for this result are as follows. The tumor cells exhibit high heterogeneity. This heterogeneity may stem from the presence of multiple concurrent tumor cell clones, each characterized by a distinct genotype. The genetic composition of tumor cells found in tumor tissue and peripheral blood may be derived from different clonal populations. Similarly, such genotypic differences may exist between metastases and primary tumors. Therefore, there may be variations in the results of genetic testing.

Increasing evidence has confirmed that ICI therapy revealed robust and sustained antitumor activity, and a favorable response in solid tumors (9, 13)with MSI-H/dMMR, including GC (10). However, approximately 50% of MSI-H cancer patients failed to respond to ICI therapy, suggesting that certain factors in the tumor microenvironment may affect the success of immune checkpoint blockade (14). In a small sample study of MSI-H gastric cancer, the level of PD-L1 expression on tumor cells or on immune cells was observed to be closely associated with survival outcomes (15). In this case, the IHC test showed PD-L1-negative, which may have partly contributed to the resistance to immunotherapy.

TMB is considered another biomarker associated with immunotherapy efficacy.

Pembrolizumab has obtained FDA approval for the treatment of solid tumors with tissue TMB-H based on the results of the KEYNOTE-158 study (16). In addition, TMB has been reported to be an important independent predictor within MSI-H mCRC treated with ICI therapy (17). However, there is currently no consistent cutoff value for TMB. An interesting study revealed substantial overlap between MSI-H and TMB-H, and the relationship between some significant mutant genes and phenotypes in 330 GC patients, providing new insights into the treatment of GC (18). In this patient, NGS results of both tumor tissue and peripheral blood showed high TMB, suggesting that other factors may be leading to immune tolerance in the tumor microenvironment.

Previous evidence has demonstrated that tumor infiltrating lymphocytes (TILs) are highly relevant to the host immune response to tumors and may predict clinical response to immunotherapy in several tumors (19–21). Cytotoxic CD8 ^+^ T cells are the main anti-tumor immune cells that clear tumor cells. CD8 ^+^ T cells have been observed to be associated with significant efficacy of immunotherapy in dMMR colorectal cancer (22). In addition, recent evidence (23) has demonstrated that a subset of CD8^+^TILs within the tumor microenvironment can recognize tumor neoantigens, suggesting an indirect anti-tumor effect. In this case, the IHC revealed that very few CD8^+^T cells were infiltrated within the tumor, and a significant number of CD8^+^T cells were filled in the stroma, which may lead to resistance to immunotherapy to some extent.

Abnormal expression or mutation of certain key genes and aberrant activation of tumor-related signaling pathways can prevent immune cell infiltration or function in the tumor microenvironment, contributing to resistance to immunotherapy (12). The mitogen-activated protein kinase (MAPK) pathway, the PTEN-PI3K-AKT signaling pathway, the WNT/b-catenin signaling pathway, interferon-gamma (IFNγ) signaling pathways, and lack of tumor antigen expression have been identified to be associated with tumor immune escape (24–29). Notably, mutations in the PTEN phosphatase domain were significantly associated with shorter survival and the presence of an immunosuppressive tumor microenvironment in MSI-H/dMMR gastrointestinal tumors patients treated with anti-PD-1 therapy (11). In addition, PTEN mutations in the phosphatase domain lead to reduced PTEN mRNA expression and loss of PTEN protein. as well as enrichment of the PI3K/AKT/mTOR and MTORC1 signaling pathways, indicative of PTEN dysfunction and possible association with anti-PD1 therapy resistance. PTEN mutation in this case may be associated with resistance to anti-PD1 therapy. It is well known that APC gene mutations play a pivotal role in tumorigenesis and progression. APC gene mutations play a crucial role in the carcinogenesis of intestinal type gastric cancer, independently of MSI status (30). Feng et al. reported that APC gene mutations may be correlated with worse response and efficacy for immunotherapy in CRC patients irrespective of MSI phenotype (31). In our case, NGS findings revealed two APC gene mutations in both the tumor tissue and peripheral blood, which may result in resistance to anti-PD1 therapy. Combined with the factors mentioned above, the tumor immune microenvironment is extremely complex, presenting great challenges in predicting the efficacy of immunotherapy. Therefore, it is necessary to comprehensively evaluate the tumor microenvironment characteristics and detailed genomic status of patients prior to ICI therapy, even for MSI-H tumors.

H. pylori colonizes the gastric mucosa and plays a significant role in the development of gastric cancer. H. pylori infection can affect gastric mucosal tumor microenvironment (TME), T cell function, and PD-L1 expression, suggesting the potential impact of H. pylori infection on immunotherapy (32). Two studies (33, 34) have shown that H. pylori infection is linked to adverse outcomes for immunotherapy in patients with advanced gastric cancer. Conversely, a large retrospective study (35) involving 10,122 patients with various cancer types indicated that H. pylori infection could be a favorable factor for immunotherapy in gastric cancer. The influence of H. pylori infection on the efficacy of immunotherapy for gastric cancer remains controversial, and the mechanisms by which H. pylori affects immunotherapy effectiveness are not yet fully understood. In our case, Helicobacter pylori negative status may have some effect on the efficacy of immunotherapy.

However, limitations of our study have to be acknowledged. Firstly, selection bias is inevitable in the present study due to a case report. Secondly, we did a small panel not gene signatures.

## Conclusions

This case has important clinical implications and provides novel insights into the treatment of solid tumors with MSI-H. The efficacy of immunotherapy for MSI-H solid tumors may be affected by mutations in key genes in tumor-related signaling pathways and the degree of CD8^+^ T cell infiltration in the tumor microenvironment.

## Data Availability

The original contributions presented in the study are included in the article/supplementary material. Further inquiries can be directed to the corresponding authors.
